# Wellness at Work for Psychiatry Trainees in Ireland: A Pilot Quality Improvement Project

**DOI:** 10.1192/j.eurpsy.2025.917

**Published:** 2025-08-26

**Authors:** P. McLoughlin, C. Dolan

**Affiliations:** 1 Sligo/Leitrim Mental Health services, Health Services Executive, Sligo; 2 The College of Psychiatrists of Ireland, Dublin; 3 Health Services Executive, Sligo, Ireland

## Abstract

**Introduction:**

Psychiatry trainees in Ireland are categorised as Non-Consultant Hospital Doctors (NCHD). Research into the wellbeing of NCHD’s has consistently highlighted difficult working conditions with the majority reporting they have been told by others they neglect their own health. The learning outcomes of the College of Psychiatrists of Ireland are majority clinical, professional and academic based with only a small focus on outcomes related to trainee wellbeing. Therefore, local and creative initiatives to support psychiatry trainee health and wellbeing are warranted.

**Objectives:**

To conduct a needs analysis amongst psychiatry trainees in the Sligo/Leitrim Mental Health Service to inform the development of a NCHD Wellness at Work Committee and assess its impact over over a six month period from January 2024 – July 2024.

**Methods:**

Following Clinical Director ethical approval, an anonymous online survey was shared with NCHD’s through email to assess their workplace wellbeing needs. Results were collected and analysed using Microsoft Excel leading to the creation and implementation of an action plan. At the end of the period, another anonymous online survey was shared through email to assess the usefulness of the project.

**Results:**

16 out of 23 local NCHD’s responded to the needs analysis.

88% identified ‘Physical Health’ as the top priority for the committee, while 75% endorsed the ‘Psychosocial work Environment’ and ‘Mental Health and Wellbeing’. The ‘Physical Work Environment’ and ‘Healthy Eating’ were chosen by 44%.

In terms of a wellness at work development program, the most frequently requested item was ‘Activities to support mental health’ (69%), followed by ‘Corporate Leisure Centre discounts’ at 63%, ‘Stress Management’ and ‘Exercise/physical activity classes’ both at 56% and ‘Access to healthy food’ and ‘Sports participation’ options both at 50%.

‘Not enough time’ was identified as the greatest barrier to participation by 69% with equal variations across preferred timing of activities.

The action plan included the improvement of the physical work environment along with the arrangement of a financial planning webinar, Human Resources Coaching, Occupational Health and Health Promotion sessions. Information was shared by email on the Cycle to Work scheme and the Living Well stress reduction program along with social events including a breakfast club, provision of time and facilitites pre-trainee teaching for social interaction and a regular social dinner.

An impact assessment survey provided 12 responses from a total requested of 16 with all 12 responding indicating a positive impact. The missing 4 responses were due to trainees moving location.

**Conclusions:**

This is, to our knowledge, the first quality improvement project to date involving the creation of an NCHD Wellness at Work Committee in Ireland.

The results will be used to inform and encourage the development of similar initiatives Deanery wide.

**Disclosure of Interest:**

None Declared

